# Prognostic Value of the Vitamin B12/C-Reactive Protein Index in Resected Stage II–III Cutaneous Melanoma: Comparison with Its Individual Components

**DOI:** 10.3390/jcm15155759

**Published:** 2026-07-23

**Authors:** Oktay Halit Aktepe, Omer Ekin, Osman Butun, Rezan Berkay Izgor, Aziz Karaoglu, Bulent Karabulut, Suayib Yalcin

**Affiliations:** 1 Department of Medical Oncology, Dokuz Eylul University, Izmir 35330, Türkiye; 2 Independent Researcher, Ankara 06800, Türkiye; 3 Department of Medical Oncology, Acibadem Izmir Kent Hospital, Izmir 35620, Türkiye; 4 Department of Internal Medicine, Faculty of Medicine, Dokuz Eylul University, Izmir 35330, Türkiye; 5 Department of Medical Oncology, Hacettepe University Cancer Institute, Ankara 06230, Türkiye

**Keywords:** C-reactive protein, cutaneous melanoma, prognostic biomarker, recurrence-free survival, vitamin B12, vitamin B12/C-reactive protein index

## Abstract

**Background**: Recurrence risk in completely resected stage II–III cutaneous melanoma remains incompletely defined by conventional staging alone. This study evaluated whether the baseline vitamin B12 (VB12)/C-reactive protein (CRP) index (BCI), a simple biomarker reflecting metabolic and inflammatory status, is associated with recurrence-free survival (RFS). **Methods**: This retrospective study included 136 patients. RFS was analyzed using Kaplan–Meier curves and Cox proportional hazards models. Receiver operating characteristic (ROC) analysis was used to determine the optimal BCI cut-off for recurrence, and a median-based cut-off was additionally examined as a sensitivity analysis. The natural logarithms (ln) of CRP, VB12, and BCI were standardized and compared in adjusted Cox models. **Results**: During a median follow-up of 62.0 months, recurrence occurred in 49 patients (36.0%). ROC analysis yielded an area under the curve of 0.66 (95% confidence interval [CI]: 0.57–0.76; *p* = 0.002), and the optimal BCI cut-off was 3386, with 65% sensitivity and 67% specificity. Patients with high BCI had significantly shorter median RFS than those with low BCI (63.5 months vs. not reached; *p* < 0.001). In multivariable analysis using the ROC-derived cut-off, higher Breslow thickness (hazard ratio [HR]: 1.10, 95% CI: 1.03–1.17; *p* = 0.002), stage III disease (HR: 2.37, 95% CI: 1.06–5.30; *p* = 0.035), and high BCI (HR: 2.39, 95% CI: 1.30–4.38; *p* = 0.005) remained independently associated with shorter RFS. The association remained significant using the median BCI cut-off (HR: 2.22, 95% CI: 1.21–4.07; *p* = 0.010). In head-to-head adjusted models, standardized ln(CRP) (HR per 1-standard-deviation [SD] increase: 1.57, 95% CI: 1.17–2.11; *p* = 0.002) and ln(BCI) (HR per 1-SD increase: 1.54, 95% CI: 1.16–2.04; *p* = 0.003) were associated with shorter RFS, whereas ln(VB12) was not (HR per 1-SD increase: 1.01, 95% CI: 0.80–1.28; *p* = 0.894). The CRP- and BCI-based models showed similar model fit. **Conclusions**: Higher baseline BCI was associated with shorter RFS after adjustment for clinicopathological factors in patients with resected stage II–III cutaneous melanoma. However, its prognostic performance was comparable to that of CRP alone, and its incremental value over CRP was not established. These findings are exploratory and require external validation before clinical application.

## 1. Introduction

Cutaneous melanoma remains a clinically important malignancy worldwide, accounting for a substantial burden of cancer incidence and mortality in the most recent global estimates [1]. The prognosis of localized and locoregionally advanced melanoma remains heterogeneous and is still primarily determined by pathological staging based on the eighth edition of the American Joint Committee on Cancer (AJCC) system [2]. Substantial variation in recurrence risk exists even within stage II and stage III disease, indicating that conventional clinicopathological variables incompletely capture the underlying biological diversity of the disease [2,3,4].

Recent advances in adjuvant systemic therapy have substantially altered the management of resected melanoma over the past decade. In patients with resected high-risk stage III/IV melanoma, adjuvant nivolumab was associated with superior recurrence-free survival (RFS) compared with ipilimumab [5]. Similarly, adjuvant pembrolizumab improved RFS compared with placebo in patients with resected stage III melanoma [6]. In patients with BRAF V600–mutant resected stage III melanoma, adjuvant dabrafenib plus trametinib improved outcomes versus placebo, supporting the role of targeted adjuvant therapy in molecularly defined disease [7]. More recently, adjuvant anti-programmed cell death protein 1 (PD-1) therapy has expanded to high-risk stage II melanoma, with randomized trials showing improved RFS for pembrolizumab in KEYNOTE-716 and nivolumab in CheckMate 76K compared with placebo [8,9]. Consistent with these advances, contemporary European Society for Medical Oncology guidance supports adjuvant systemic therapy as a standard consideration in the management of high-risk resected disease [10]. Despite progress in adjuvant therapy, the routine management of resected stage II–III melanoma still requires better tools to minimize unnecessary treatment exposure and to improve risk identification among high-risk patients. This has sustained interest in pragmatic biomarkers that are inexpensive, reproducible, and readily implementable in clinical practice alongside AJCC staging.

Systemic inflammation is closely linked to carcinogenesis and tumor progression through its effects on immune regulation, angiogenesis, and metastatic potential [11]. As a readily measurable acute-phase reactant, C-reactive protein (CRP) has been extensively evaluated as a clinically practical biomarker reflecting cancer-related systemic inflammation and tumor–host interactions [12,13]. In melanoma, elevated baseline inflammatory markers, including CRP, have been associated with poorer survival outcomes across cohorts and immunotherapy trial datasets, supporting the prognostic relevance of the host inflammatory state [14,15,16,17]. Likewise, a melanoma-focused meta-analysis found that higher CRP levels were associated with poorer overall survival (OS) and progression-related endpoints across several studies, further underscoring its value as a robust prognostic signal at the population level [13].

Vitamin B12 (VB12) has been evaluated mainly for deficiency states; however, hypercobalaminemia has increasingly been associated with underlying cancer and adverse clinical outcomes in large population-based analyses [18]. Mechanistically, elevated VB12 may reflect cancer- and inflammation-related systemic disturbances rather than nutritional sufficiency alone [19].

The VB12/CRP index (BCI), obtained by multiplying serum VB12 by CRP, was developed as a composite biomarker that integrates metabolic-nutritional status with systemic inflammation. In advanced cancer and palliative settings, higher BCI was associated with markedly shorter survival [20,21,22]. In older cancer populations, elevated BCI has been associated with clinically relevant adverse outcomes, supporting its potential value for risk stratification [23]. However, the prognostic value of BCI in resected stage II–III cutaneous melanoma remains uncertain, despite heterogeneous recurrence risk and the need for improved biomarker-informed stratification. Therefore, this study aimed to evaluate the association between baseline BCI and RFS in patients with completely resected stage II–III cutaneous melanoma and to compare its prognostic performance with those of its individual components, CRP and VB12.

## 2. Materials and Methods

### 2.1. Study Design and Patients

This retrospective multicenter cohort study included all patients with histologically confirmed cutaneous melanoma who met the predefined eligibility criteria and were identified in the available medical records at three participating sites between January 2010 and January 2025: the Department of Medical Oncology, Dokuz Eylul University, Izmir, Türkiye; the Department of Medical Oncology, Acibadem Izmir Kent Hospital, Izmir, Türkiye; and a private oncology practice in Ankara, Türkiye. Patients who had received their initial treatment at other institutions and were subsequently referred to one of the participating sites for follow-up were also eligible. Comprehensive screening logs covering all patients with cutaneous melanoma evaluated at these sites during the study period were not maintained; therefore, the total source population and sequential reason-specific exclusion counts could not be reliably reconstructed retrospectively. All included patients had AJCC stage II or III disease and underwent curative-intent surgery with R0 resection, defined as complete excision with no microscopic residual disease. We included patients aged 18 years or older with histologically confirmed stage II–III cutaneous melanoma who underwent complete surgical resection with curative intent and had available data on baseline serum VB12 and CRP levels measured within three months before surgery; we excluded those with (1) stage I or stage IV disease, (2) non-cutaneous melanoma subtypes (including mucosal or uveal melanoma), (3) incomplete resection, (4) missing baseline VB12 or CRP data within the predefined preoperative period, (5) insufficient clinicopathological or follow-up data for recurrence assessment, and (6) concurrent conditions that could substantially affect VB12 or CRP levels independently of melanoma, including active infection, acute inflammatory disorders, significant hepatic dysfunction, hematologic disorders, or recent VB12 supplementation, when such data were available. Baseline demographic, pathological, and treatment-related data were retrieved from institutional medical records. The collected variables included age, sex, Breslow thickness, Clark level, mitotic rate, ulceration status, tumor stage, tumor location, adjuvant treatment status, serum VB12 level, and serum CRP level.

The BCI was calculated by multiplying the serum VB12 concentration (pg/mL) by the serum CRP concentration (mg/L), using the baseline laboratory values recorded for each patient. Baseline VB12 and CRP measurements were obtained within three months before surgery as part of routine clinical care at the three participating sites. Although all results were recorded using common units, namely pg/mL for VB12 and mg/L for CRP, the assay platforms, reagents, and exact timing of blood sampling within the predefined preoperative window were not fully standardized across sites or throughout the study period. To evaluate the prognostic performance of BCI, patients were classified using two different approaches. First, an optimal cut-off value was determined by receiver operating characteristic (ROC) curve analysis for RFS. Second, an additional analysis was performed using the cohort median BCI value as an alternative threshold. According to these approaches, patients were stratified into low- and high-BCI groups for comparative and survival analyses.

### 2.2. Statistical Analysis

Categorical variables were summarized as numbers and percentages, whereas continuous variables were expressed as median and interquartile range (IQR). Comparisons between low- and high-BCI groups were performed using the chi-square test or Fisher’s exact test for categorical variables, and the Mann–Whitney U test for continuous variables, as appropriate. RFS was defined as the time from curative-intent surgery to the date of first documented recurrence or last disease assessment. Patients without recurrence were censored at the date of last disease assessment. OS was not analyzed because RFS was the primary endpoint of interest and was considered the outcome most directly reflecting postoperative melanoma recurrence. In addition, OS would have been susceptible to confounding by heterogeneous post-recurrence treatments and non-melanoma-related mortality over the prolonged study period.

The discriminative ability of BCI for recurrence status was evaluated using ROC curve analysis, and the optimal cut-off value was determined according to the highest combined sensitivity and specificity. Survival curves were generated using the Kaplan–Meier method and compared using the log-rank test. In addition to the overall cohort analysis, exploratory stage-stratified Kaplan–Meier analyses were performed.

To investigate factors associated with RFS, univariable Cox proportional hazards regression analyses were initially conducted for selected clinicopathological variables. Variables considered clinically relevant and those associated with RFS at a significance threshold of *p* ≤ 0.20 in univariable analysis were subsequently entered into multivariable Cox regression models. To assess the robustness of the findings, separate multivariable models were constructed using the ROC-derived BCI cut-off and the cohort median BCI value as an alternative threshold.

BCI was also analyzed as a continuous variable after natural logarithmic transformation [ln(BCI)] to reduce information loss related to dichotomization. To further explore the shape of the association between ln(BCI) and RFS, ln(BCI) was categorized into tertiles. A multivariable Cox regression model was fitted using the lowest tertile as the reference category, with adjustment for age, Breslow thickness, tumor stage, and adjuvant treatment. In a separate model, tertiles were entered as an ordinal variable coded as 1, 2, and 3 to assess the linear trend across increasing ln(BCI) categories.

To explore the temporal stability of the association between BCI and RFS, the cohort was divided according to the median calendar date of cohort entry into an early period, defined as entry on or before 31 March 2021, and a late period, defined as entry after 31 March 2021. A multivariable Cox regression model including standardized ln(BCI), calendar period, and an ln(BCI) × calendar-period interaction term was fitted, with adjustment for age, Breslow thickness, tumor stage, and adjuvant treatment.

For the head-to-head comparison, CRP, VB12, and BCI were ln-transformed and standardized to a mean of 0 and a standard deviation (SD) of 1. Separate Cox models were constructed by adding standardized ln(CRP), ln(VB12), or ln(BCI) to a clinical model including age, Breslow thickness, tumor stage, and adjuvant treatment. An additional model included standardized ln(CRP) and ln(VB12) simultaneously. Model fit was assessed using the −2 log likelihood and Akaike information criterion (AIC). Nested models were compared using likelihood-ratio tests to evaluate the improvement obtained by adding standardized ln(BCI) to the clinical model and standardized ln(VB12) to the CRP-based model.

Harrell’s concordance index (C-index) was calculated for CRP, VB12, and BCI as continuous markers. Because all biomarkers were measured in the same patients, paired comparisons accounting for the covariance between estimates were performed. Internal validation was conducted using 1000 bootstrap resamples, and optimism-corrected C-indices were reported.

All tests were two-sided, and *p* < 0.05 was considered statistically significant. Statistical analyses were performed using the Statistical Package for the Social Sciences (SPSS) version 27.0 (IBM Corp., Armonk, NY, USA), whereas ROC curve analyses, Kaplan–Meier survival analyses, C-index analyses, paired comparisons, and bootstrap internal validation were conducted in R version 4.6.0 (R Foundation for Statistical Computing, Vienna, Austria).

## 3. Results

### 3.1. Baseline Characteristics of the Overall Cohort and BCI Subgroups

**Figure 1:**
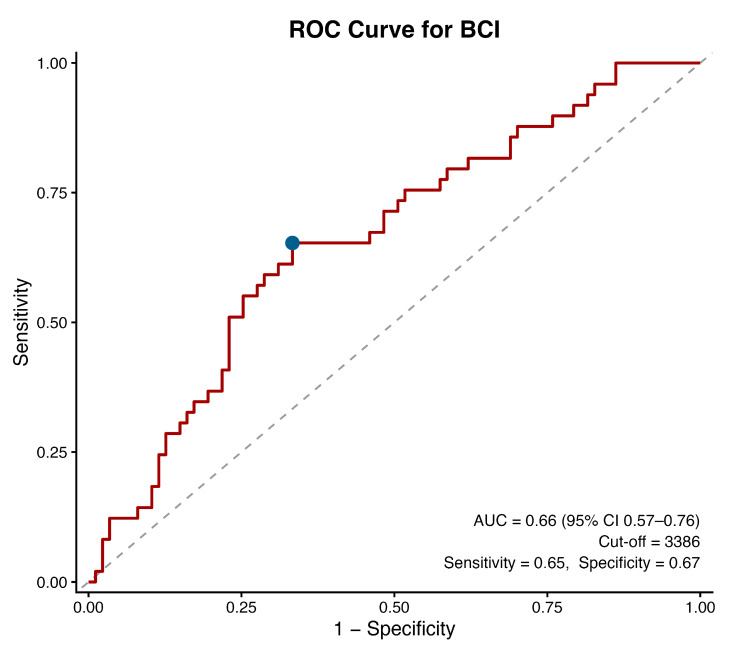
ROC curve of BCI for discrimination of recurrence status in resected stage II–III cutaneous melanoma. Abbreviations: AUC: area under the curve; BCI: vitamin B12/C-reactive protein index; CI: confidence interval; ROC: receiver operating characteristic.

A total of 136 patients were included in the study. The ROC analysis for recurrence status yielded an area under the curve of 0.66 (95% confidence interval [CI]: 0.57–0.76; *p* = 0.002) for BCI. The best cut-off value was determined as 3386, providing 65% sensitivity and 67% specificity (Figure 1). According to BCI stratification, 74 patients (54.4%) were classified into the low-BCI group and 62 patients (45.6%) into the high-BCI group. Baseline demographic and clinicopathological characteristics according to BCI status are summarized in Table 1. In the overall cohort, the median age was 59 years (IQR, 49–69), and most patients were male (61%). The median Breslow thickness was 4.7 mm (IQR, 2.8–8.3), while the median mitotic rate was 8 (IQR, 4–14). With regard to pathological characteristics, Clark level IV–V was observed in 76.5% of patients, ulceration was present in 58.1%, and stage III disease was identified in 58.1% of the cohort. In addition, 38.2% of patients had received adjuvant therapy.

When patients were compared according to BCI status, baseline demographic and clinicopathological features were generally well balanced between the low- and high-BCI groups. The median age was 58 years (IQR, 46–69) in the low-BCI group and 62 years (IQR, 49–69) in the high-BCI group (*p* = 0.238). Male sex was slightly more frequent in the high-BCI group than in the low-BCI group (64.5% vs. 58.1%), although this difference was not statistically significant (*p* = 0.445). Similarly, Breslow thickness [4.2 (IQR, 2.2–7.8) vs. 5.1 (IQR, 3.3–9.3); *p* = 0.212] and mitotic rate [6 (IQR, 3–14) vs. 8 (IQR, 4–14); *p* = 0.304] were comparable between the low- and high-BCI groups. No significant between-group differences were observed for Clark level distribution (*p* = 0.519), ulceration status (*p* = 0.996), tumor stage (*p* = 0.298), tumor location (*p* = 0.471), or receipt of adjuvant therapy (*p* = 0.418). Overall, these findings indicate that the two BCI subgroups were largely similar with respect to baseline clinicopathological characteristics.

In the overall cohort, the median VB12 level was 325.7 pg/mL (IQR, 247.3–458.0). The median VB12 levels were 283.2 pg/mL (IQR, 189.2–369.5) in the low-BCI group and 439.6 pg/mL (IQR, 286.5–583.6) in the high-BCI group. The median CRP level was 9 mg/L (IQR, 5.2–20.9) in the overall cohort, 5.5 mg/L (IQR, 3.6–8.7) in the low-BCI group, and 19.6 mg/L (IQR, 10.5–37.1) in the high-BCI group.

### 3.2. RFS According to BCI Stratification

**Figure 2:**
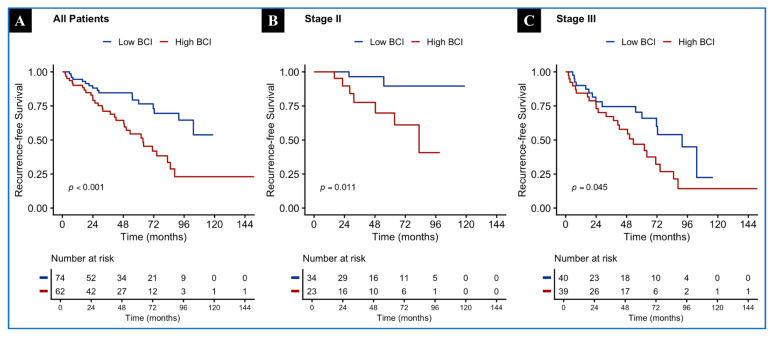
Kaplan–Meier curves for RFS according to ROC-derived BCI stratification in the overall cohort (**A**), stage II subgroup (**B**), and stage III subgroup (**C**). Abbreviations: BCI: vitamin B12/C-reactive protein index; RFS: recurrence-free survival; ROC: receiver operating characteristic.

The median follow-up was 62.0 months (95% CI: 46.7–77.3), and recurrence was documented in 49 of 136 patients (36.0%). Of the 49 recurrence events, 9 occurred among 57 patients with stage II disease and 40 among 79 patients with stage III disease. Kaplan–Meier analysis demonstrated a significant difference in RFS between the BCI groups in the overall cohort (*p* < 0.001, Figure 2A). The median RFS was 63.5 months (95% CI: 43.2–83.7) in the high-BCI group, whereas it was not reached in the low-BCI group. Exploratory stage-stratified Kaplan–Meier analyses were also performed. In stage II disease, the median RFS was not reached in the low-BCI group, whereas it was 82.8 months (95% CI: 47.9–117.8) in the high-BCI group (*p* = 0.011, Figure 2B). In stage III disease, the median RFS was 91.7 months (95% CI: 60.1–123.4) in the low-BCI group and 53.3 months (95% CI: 31.3–75.3) in the high-BCI group (*p* = 0.045, Figure 2C).

**Figure 3:**
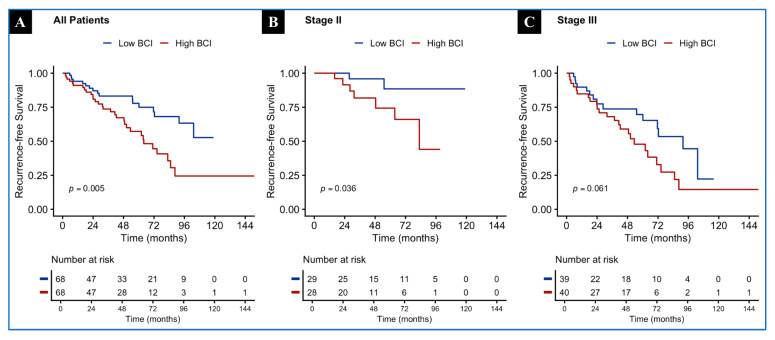
Kaplan–Meier curves for RFS according to median-based BCI stratification in the overall cohort (**A**), stage II subgroup (**B**), and stage III subgroup (**C**). Abbreviations: BCI: vitamin B12/C-reactive protein index; RFS: recurrence-free survival.

Kaplan–Meier analysis using the cohort median BCI cut-off of 3026 demonstrated a significant difference in RFS in the overall cohort (*p* = 0.005, Figure 3A). Patients with high BCI had a median RFS of 64.0 months (95% CI: 43.1–84.8), whereas the median RFS was not reached in those with low BCI. Exploratory stage-stratified analyses were also performed. In patients with stage II disease, the median RFS was not reached in the low-BCI group, whereas it was 82.8 months (95% CI: 48.9–116.7) in the high-BCI group (*p* = 0.036, Figure 3B). In patients with stage III disease, the median RFS was 91.7 months (95% CI: 60.0–123.5) in the low-BCI group and 53.3 months (95% CI: 34.9–71.7) in the high-BCI group; however, the difference did not reach statistical significance (*p* = 0.061, Figure 3C).

### 3.3. Cox Regression Analyses and Head-to-Head Biomarker Comparison

As presented in Table 2, univariable Cox regression analysis showed that Breslow thickness, stage III disease, lack of adjuvant therapy, and high BCI defined by the ROC-derived cut-off of 3386 were significantly associated with shorter RFS. Increased Breslow thickness was associated with a higher risk of recurrence (hazard ratio [HR]: 1.13, 95% CI: 1.07–1.19; *p* < 0.001), as were stage III disease (HR: 3.57, 95% CI: 1.73–7.39; *p* < 0.001), absence of adjuvant treatment (HR: 2.19, 95% CI: 1.24–3.89; *p* = 0.007), and high BCI (HR: 2.63, 95% CI: 1.45–4.78; *p* = 0.001). In the multivariable model using the ROC-derived BCI cut-off of 3386, Breslow thickness (HR: 1.10, 95% CI: 1.03–1.17; *p* = 0.002), stage III disease (HR: 2.37, 95% CI: 1.06–5.30; *p* = 0.035), and high BCI (HR: 2.39, 95% CI: 1.30–4.38; *p* = 0.005) remained independently associated with shorter RFS, whereas age and adjuvant therapy were not statistically significant. Similarly, in the multivariable model based on the median BCI cut-off of 3026, Breslow thickness (HR: 1.10, 95% CI: 1.03–1.17; *p* = 0.002), stage III disease (HR: 2.37, 95% CI: 1.06–5.31; *p* = 0.035), and high BCI (HR: 2.22, 95% CI: 1.21–4.07; *p* = 0.010) remained independently associated with shorter RFS.

As presented in Table 3, to further explore the shape of the association between ln(BCI) and RFS, ln(BCI) was categorized into tertiles. In the multivariable Cox model adjusted for age, Breslow thickness, tumor stage, and adjuvant treatment, the overall association between ln(BCI) tertiles and RFS was statistically significant (*p* = 0.004). Compared with the lowest tertile, the intermediate tertile was associated with a numerically higher recurrence risk, although this difference did not reach statistical significance (HR: 1.81, 95% CI: 0.78–4.19; *p* = 0.162). In contrast, the highest tertile was significantly associated with an increased recurrence risk (HR: 3.44, 95% CI: 1.61–7.35; *p* = 0.001). When ln(BCI) tertiles were entered as an ordinal variable, each one-tertile increase was independently associated with a higher recurrence risk (HR: 1.86, 95% CI: 1.28–2.69; *p* for trend < 0.001), supporting a progressive increase in recurrence risk across the ln(BCI) distribution.

To assess the temporal stability of the association between BCI and RFS, an exploratory interaction analysis was performed after dividing the cohort according to the median calendar date of cohort entry. In the multivariable Cox model adjusted for age, Breslow thickness, tumor stage, and adjuvant treatment, the HR per 1-SD increase in standardized ln(BCI) was 1.80 in the early period (95% CI: 1.28–2.54; *p* < 0.001). The corresponding HR was 1.08 in the late period (95% CI: 0.63–1.86; *p* = 0.774). The difference between the two period-specific HRs was not statistically significant (*p* for interaction = 0.121).

Head-to-head Cox models adjusted for age, Breslow thickness, tumor stage, and adjuvant treatment were used to compare the prognostic associations of CRP, VB12, and BCI with RFS (Table 4). Standardized ln(CRP) was independently associated with shorter RFS (HR per 1-SD increase: 1.57, 95% CI: 1.17–2.11; *p* = 0.002), whereas standardized ln(VB12) was not (HR per 1-SD increase: 1.01, 95% CI: 0.80–1.28; *p* = 0.894). Standardized ln(BCI) was also independently associated with shorter RFS (HR per 1-SD increase: 1.54, 95% CI: 1.16–2.04; *p* = 0.003), and its addition to the clinical model significantly improved model fit (likelihood-ratio χ^2^ = 9.348, degrees of freedom [df] = 1; *p* = 0.002). When standardized ln(CRP) and ln(VB12) were entered simultaneously, ln(CRP) remained significantly associated with shorter RFS (HR: 1.64, 95% CI: 1.21–2.22; *p* = 0.001), whereas ln(VB12) remained non-significant (HR: 1.14, 95% CI: 0.89–1.45; *p* = 0.277). The addition of ln(VB12) to the CRP-based model did not significantly improve model fit (likelihood-ratio χ^2^ = 1.207, df = 1; *p* = 0.272). The CRP- and BCI-based models had comparable AIC values (375.885 and 376.739, respectively).

Harrell’s C-index was calculated for CRP, VB12, and BCI as continuous markers. Because all three biomarkers were measured in the same patients, each marker was refitted within 1000 bootstrap resamples to preserve the covariance among the estimates. Optimism-corrected C-indices and their pairwise differences were subsequently calculated. The optimism-corrected C-index was 0.615 for CRP, 0.617 for BCI, and 0.518 for VB12. None of the pairwise differences in discrimination was statistically significant. The difference between BCI and CRP was ΔC = 0.002 (95% CI, −0.090 to 0.060; *p* = 0.96), the difference between BCI and VB12 was ΔC = 0.099 (95% CI, −0.044 to 0.208; *p* = 0.14), and the difference between CRP and VB12 was ΔC = 0.097 (95% CI, −0.047 to 0.218; *p* = 0.18). BCI and CRP were strongly correlated (Spearman’s ρ = 0.80).

## 4. Discussion

In this retrospective cohort of patients with completely resected stage II–III cutaneous melanoma, higher baseline BCI was associated with shorter RFS across ROC-derived and median-based cut-offs, continuous ln-transformed analysis, and exploratory tertile-based analyses. No significant variation in this association was observed between the early and late study periods. However, head-to-head analyses using standardized effect estimates showed comparable prognostic associations and model performance for ln(BCI) and ln(CRP), whereas ln(VB12) was not independently associated with RFS. Moreover, BCI did not significantly improve optimism-corrected discrimination over CRP alone. Thus, although BCI was independently associated with recurrence risk, its incremental prognostic value beyond CRP was not demonstrated.

The stage-stratified findings should also be interpreted cautiously. Although the ROC-derived BCI cut-off was associated with RFS in both stage II and stage III disease, only 9 recurrence events occurred among patients with stage II disease, substantially limiting the precision and stability of this subgroup estimate. Using the median BCI cut-off, the difference remained statistically significant in stage II disease, whereas the stage III comparison did not reach statistical significance despite a numerical separation of the survival curves. These variations may reflect limited statistical power rather than a true stage-specific difference in the prognostic association of BCI. Accordingly, the subgroup analyses should be regarded as exploratory and do not establish that the prognostic performance of BCI is consistent across individual disease stages.

From a biological perspective, the association between elevated BCI and inferior RFS is plausible. Systemic inflammation is increasingly recognized as a key driver of cancer development and progression by shaping antitumor immune surveillance, cytokine-mediated signaling networks, extracellular matrix remodeling, and metastatic dissemination [11,24]. CRP is an accessible downstream marker of inflammatory activity and may serve as a surrogate for host–tumor interactions [25,26]. In melanoma, inflammatory mediators, including CRP, have shown prognostic relevance in immune checkpoint inhibitor cohorts and clinical trial datasets, in which higher baseline CRP and related inflammatory markers were associated with shorter survival [16,27]. A broader synthesis of the melanoma literature also showed that elevated CRP was associated with inferior survival across heterogeneous cohorts, supporting a consistent association between systemic inflammation and prognosis [13]. Although CRP has been studied predominantly in advanced disease and immunotherapy settings [16,28], the present study extends the inflammation–outcome association to resected stage II–III melanoma, where recurrence risk remains clinically important.

High plasma VB12 levels are frequently observed in clinical practice and have been associated with an increased risk of cancer in several human studies [18,29,30]. This association may be particularly evident in patients with markedly elevated VB12 concentrations or persistently high levels on longitudinal assessment [18,29]. Among patients with established malignancy, elevated circulating VB12 has been associated with poorer survival, suggesting that hypercobalaminemia may reflect more advanced or biologically aggressive disease [31]. However, current evidence does not support a direct cancer-promoting role for VB12 itself. Instead, elevated circulating VB12 likely reflects disrupted cobalamin homeostasis in malignancy, potentially related to inflammation, hepatic involvement, or greater disease burden, rather than a direct tumor-promoting effect [19,30,32]. Therefore, elevated VB12 likely reflects underlying disease activity and should not be interpreted as a causal factor in carcinogenesis.

The median VB12 concentration in the present cohort was 325.7 pg/mL and did not indicate cohort-wide hypercobalaminemia. Accordingly, the findings should not be interpreted as evidence that elevated VB12 itself was the principal prognostic factor. Although VB12 concentrations were higher in the high-BCI group, standardized ln(VB12) was not independently associated with RFS, and its addition to the CRP-based model did not significantly improve model fit. These findings indicate that the association between BCI and recurrence risk primarily reflected its inflammatory component rather than additional prognostic information contributed by VB12.

Importantly, BCI should not be regarded as a novel or melanoma-specific construct, but rather as a composite biomarker that has been previously investigated in other oncologic settings. In a confirmatory study of patients with advanced cancer, Kelly et al. reported that increasing BCI categories were associated with progressively shorter survival, and that a BCI value above 40,000 identified a subgroup with particularly poor prognosis [20]. In line with these observations, elevated BCI has also been reported as a simple adverse prognostic indicator in patients with metastatic renal cell carcinoma treated with first-line targeted therapy [33], suggesting that this composite index may retain prognostic relevance across different tumor types and treatment settings. This concept has also been extended to older adults with cancer. Couderc et al. reported that elevated BCI was associated with both higher short-term mortality and unplanned hospitalization in older patients with cancer, and suggested that BCI could be integrated into comprehensive geriatric assessment to improve pretreatment risk stratification [23]. Similarly, Montegut et al. demonstrated in a large cohort of cancer patients aged 70 years and older that a BCI > 10,000 was associated with shorter survival and markers of pre-existing geriatric vulnerability, including impaired functional status and malnutrition [34]. Taken together, these studies reinforce the prognostic significance of BCI across heterogeneous oncology populations. At the same time, our melanoma-specific findings suggest that absolute BCI cut-offs may not be directly generalizable across different clinical settings. In particular, BCI cut-offs derived from advanced or frailer cancer populations may be higher than those relevant to resected stage II–III melanoma because of differences in inflammatory burden, VB12 distribution, and disease biology.

From a clinical perspective, BCI has the practical advantage of being easily calculated from routinely available serum VB12 and CRP measurements, without the need for additional tissue sampling or specialized testing. However, in the present study, BCI did not show better prognostic performance than CRP alone. Therefore, it should not be considered a replacement for CRP, AJCC staging, or established clinicopathological risk factors. Its potential value may lie in offering a simple reflection of systemic inflammatory status, but whether it provides clinically meaningful additional information for postoperative risk assessment, follow-up planning, or treatment decisions needs to be confirmed in independent cohorts. BCI should also be interpreted as a prognostic rather than predictive biomarker, since this study did not assess whether it identifies patients who derive greater benefit from adjuvant anti-PD-1 or combined BRAF and mitogen-activated protein kinase kinase (MEK) inhibitor therapy.

This study has several limitations that should be acknowledged. A primary limitation of this study is the absence of external validation. The optimal BCI threshold was derived and evaluated within the same relatively small cohort, introducing a substantial risk of optimism and overfitting and potentially limiting its transportability to other populations. Although the consistency of the findings using a median-based cut-off provides some evidence of robustness, this sensitivity analysis and the internal bootstrap validation do not replace validation in an independent cohort. Second, its retrospective design and limited number of participating centers introduce the possibility of selection bias, residual confounding, and heterogeneity in clinical management and laboratory timing. In addition, comprehensive screening registries covering all patients with cutaneous melanoma evaluated at the three participating sites were not available. Consequently, the total source population and the numbers excluded for individual reasons could not be quantified, limiting assessment of cohort representativeness and introducing the possibility of additional selection bias related to incomplete routine clinical documentation. Third, both components of BCI are susceptible to non-tumor-related influences. CRP is a non-specific inflammatory marker and may be elevated in the presence of infection, autoimmune disorders, or other inflammatory conditions, while serum VB12 levels can be affected by supplementation, liver dysfunction, hematologic disorders, and altered binding protein dynamics. Accordingly, the observed prognostic association may not be entirely cancer-specific. Fourth, the sample size was modest, and the number of recurrence events was relatively limited, which restrict statistical power and increase the risk of model instability, particularly in subgroup analyses. Fifth, laboratory measurements were obtained as part of routine clinical care across three participating sites over a prolonged study period. Assay platforms and reagents were not standardized across sites or over time, and the exact timing of blood sampling varied within the predefined three-month preoperative window. Consequently, CRP, VB12, and the derived BCI values may not have uniformly represented the same immediate preoperative biological state. This inter-site, temporal, and sampling-time heterogeneity may have introduced measurement error, affected the observed associations, and limited the reproducibility and generalizability of the derived BCI cut-offs. Finally, because this study was not designed to assess treatment interaction, no conclusions can be drawn regarding the predictive value of BCI for benefit from adjuvant immunotherapy or targeted therapy. Therefore, the present findings should be interpreted as hypothesis-generating and warrant confirmation in larger, prospective, multicenter studies.

## 5. Conclusions

In patients with completely resected stage II–III cutaneous melanoma, higher baseline BCI was consistently associated with shorter RFS across several analytical approaches. Nevertheless, its prognostic performance was comparable to that of CRP alone, and no incremental prognostic value over CRP was demonstrated. Given the retrospective design, heterogeneity in laboratory methods and preoperative sampling times, modest sample size, limited number of recurrence events, and absence of external validation, these findings should be regarded as hypothesis-generating and do not yet support the routine clinical use of BCI. Prospective studies with standardized sampling and independent external validation are required.

## Figures and Tables

**Table 1:** Baseline demographic and clinicopathological characteristics according to BCI subgroups.

Characteristics	All Patients (*n* = 136)	Low BCI (*n* = 74, 54.4%)	High BCI (*n* = 62, 45.6%)	*p* Value
Age, years, median (IQR)	59 (49–69)	58 (46–69)	62 (49–69)	0.238
Sex				0.445
Female	53 (39%)	31 (41.9%)	22 (35.5%)	
Male	83 (61%)	43 (58.1%)	40 (64.5%)	
Breslow thickness, mm, median (IQR)	4.7 (2.8–8.3)	4.2 (2.2–7.8)	5.1 (3.3–9.3)	0.212
Clark level				0.519
II–III	32 (23.5%)	19 (25.7%)	13 (21%)	
IV–V	104 (76.5%)	55 (74.3%)	49 (79%)	
Mitotic rate, per mm^2^, median (IQR)	8 (4–14)	6 (3–14)	8 (4–14)	0.304
Ulceration				0.996
No	57 (41.9%)	31 (41.9%)	26 (41.9%)	
Yes	79 (58.1%)	43 (58.1%)	36 (58.1%)	
Tumor stage				0.298
II	57 (41.9%)	34 (45.9%)	23 (37.1%)	
III	79 (58.1%)	40 (54.1%)	39 (62.9%)	
Tumor location				0.471
Trunk	47 (34.6%)	29 (39.2%)	18 (29%)	
Extremity	53 (39%)	27 (36.5%)	26 (41.9%)	
Head and neck	36 (26.5%)	18 (24.3%)	18 (29%)	
Adjuvant therapy				0.418
No	84 (61.8%)	48 (64.9%)	36 (58.1%)	
Yes	52 (38.2%)	26 (35.1%)	26 (41.9%)	

Abbreviations: BCI: vitamin B12/C-reactive protein index; IQR: interquartile range.

**Table 2:** Univariable and multivariable Cox regression analyses for RFS using the ROC-derived and median BCI cut-offs.

Variable	Univariable Analysis ^a^		Multivariable Analysis ROC-Derived Cut-Off ^a^		Multivariable Analysis Median Cut-Off ^b^	
	HR (95% CI)	*p* Value	HR (95% CI)	*p* Value	HR (95% CI)	*p* Value
Age, years	0.98 (0.96–1.00)	0.065	0.98 (0.95–1.00)	0.095	0.98 (0.96–1.00)	0.108
Sex (male vs. female)	1.14 (0.62–2.09)	0.656	NA	NA	NA	NA
Breslow thickness	1.13 (1.07–1.19)	<0.001	1.10 (1.03–1.17)	0.002	1.10 (1.03–1.17)	0.002
Mitotic rate	1.00 (0.98–1.01)	0.966	NA	NA	NA	NA
Stage (III vs. II)	3.57 (1.73–7.39)	<0.001	2.37 (1.06–5.30)	0.035	2.37 (1.06–5.31)	0.035
Adjuvant therapy (no vs. yes)	2.19 (1.24–3.89)	0.007	1.14 (0.60–2.17)	0.638	1.19 (0.63–2.26)	0.588
BCI (high vs. low)	2.63 (1.45–4.78)	0.001	2.39 (1.30–4.38)	0.005	2.22 (1.21–4.07)	0.010

Abbreviations: BCI: vitamin B12/C-reactive protein index; CI: confidence interval; HR: hazard ratio; NA: not applicable; RFS: recurrence-free survival; ROC: receiver operating characteristic. ^a^ For the univariable analysis and the ROC-derived multivariable model, BCI (high vs. low) was defined using the ROC-derived cut-off of 3386. ^b^ For the median-based multivariable model, BCI (high vs. low) was defined using the cohort median cut-off of 3026.

**Table 3:** Exploratory multivariable Cox regression analyses evaluating the association between ln(BCI) tertiles and RFS.

**Panel (A).** ln(BCI) tertiles entered as a categorical variable		
**Variable**	**HR (95% CI)**	***p* Value **
Age, years	0.97 (0.95–0.99)	0.040
Breslow thickness	1.09 (1.03–1.16)	0.003
Stage (III vs. II)	2.42 (1.07–5.47)	0.033
Adjuvant treatment (no vs. yes)	1.16 (0.60–2.23)	0.644
ln(BCI) tertiles, overall	—	0.004
Tertile 2 vs. tertile 1	1.81 (0.78–4.19)	0.162
Tertile 3 vs. tertile 1	3.44 (1.61–7.35)	0.001
**Panel (B).** ln(BCI) tertiles entered as an ordinal variable		
**Variable**	**HR (95% CI)**	***p* Value **
Age, years	0.97 (0.95–0.99)	0.040
Breslow thickness	1.09 (1.03–1.16)	0.003
Stage (III vs. II)	2.42 (1.07–5.46)	0.033
Adjuvant treatment (no vs. yes)	1.16 (0.61–2.23)	0.640
ln(BCI), per one-tertile increase	1.86 (1.28–2.69)	<0.001

Abbreviations: BCI: vitamin B12/C-reactive protein index; CI: confidence interval; HR: hazard ratio; ln: natural logarithm; RFS: recurrence-free survival. Tertile 1 represents the lowest third, tertile 2 the middle third, and tertile 3 the highest third of the ln(BCI) distribution. Tertile 1 was used as the reference category in Panel (A). Panel (B) represents a separate model in which tertiles were coded ordinally as 1, 2, and 3; its *p* value therefore represents the test for trend. Both models were adjusted for age, Breslow thickness, tumor stage, and adjuvant treatment.

**Table 4:** Head-to-head adjusted Cox models comparing CRP, VB12, and BCI for RFS.

Model	Biomarker	HR per 1-SD Increase (95% CI)	*p* Value	−2 Log Likelihood	AIC
Clinical model	—	—	—	376.087	384.087
Clinical model + ln(CRP)	ln(CRP)	1.57 (1.17–2.11)	0.002	365.885	375.885
Clinical model + ln(VB12)	ln(VB12)	1.01 (0.80–1.28)	0.894	376.069	386.069
Clinical model + ln(CRP) + ln(VB12)	ln(CRP)	1.64 (1.21–2.22)	0.001	364.678	376.678
	ln(VB12)	1.14 (0.89–1.45)	0.277		
Clinical model + ln(BCI)	ln(BCI)	1.54 (1.16–2.04)	0.003	366.739	376.739

**Abbreviations:** AIC: Akaike information criterion; BCI: vitamin B12/C-reactive protein index; CI: confidence interval; CRP: C-reactive protein; HR: hazard ratio; ln: natural logarithm; RFS: recurrence-free survival; SD: standard deviation; VB12: vitamin B12.

## Data Availability

The datasets generated and/or analyzed during the current study are available from the corresponding author on reasonable request, subject to patient privacy and ethical considerations.

## Abbreviations

The following abbreviations are used in this manuscript:

AJCC	American Joint Committee on Cancer
AIC	Akaike information criterion
AUC	area under the curve
BCI	vitamin B12/C-reactive protein index
CI	confidence interval
C-index	concordance index
CRP	C-reactive protein
df	degrees of freedom
HR	hazard ratio
IQR	interquartile range
ln	natural logarithm
MEK	mitogen-activated protein kinase kinase
NA	not applicable
OS	overall survival
PD-1	programmed cell death protein 1
RFS	recurrence-free survival
ROC	receiver operating characteristic
SD	standard deviation
SPSS	Statistical Package for the Social Sciences
VB12	vitamin B12
